# Mannan oligosaccharides improve the fur quality of raccoon dogs by regulating the gut microbiota

**DOI:** 10.3389/fmicb.2023.1324277

**Published:** 2023-12-15

**Authors:** Chongshan Yuan, Lili Ren, Rui Sun, Xianghong Yun, Xiao Zang, Aiwu Zhang, Min Wu

**Affiliations:** ^1^College of Animal Science and Technology, Jilin Agricultural University, Changchun, China; ^2^Animal Husbandry Research Institute, Changchun Academy of Agricultural Science, Changchun, China

**Keywords:** mannan oligosaccharides, raccoon dogs, fur quality, antioxidant, immunity, gut microbiome

## Abstract

**Introduction:**

Adding antibiotics to animal basal diets can improve growth and production performance. However, the use of antibiotics poses a potential threat to public health safety.

**Methods:**

The study was conducted to investigate the effects of different levels of mannan oligosaccharides (MOS) on the fur quality, nutrient apparent digestibility, serum immunity, antioxidant status, intestinal morphology, and gut microbiota of fur-growing raccoon dogs. Divide 24 male raccoon dogs (120 ± 5 d) of similar weight (5.01 ± 0.52 kg) into 4 groups randomly. Add 0, 0.05, 0.1, and 0.2% MOS to the basal diets of groups C, L, M, and H, respectively.

**Results:**

Compared to the C group, the addition of 0.05% and 0.1% MOS in the diet increased the apparent digestibility of crude protein (CP), Underfur length (UL), Guard hair length (GL), immunoglobulin A (IgA), immunoglobulin G (IgG), and immunoglobulin M (IgM) levels in the serum (*p* < 0.05); Under the dosage of 0.05 % MOS, the activities of Superoxide Dismutase (SOD) and catalase (CAT) increased (*p* < 0.05). Compared to the C group, adding 0.05% MOS significantly increased the VH/CD of the duodenum and ileum, while also increasing the VH and CD of the jejunum (*p* < 0.05). Through Spearman correlation analysis of the gut microbiota, it was found that MOS can improve fur quality by reducing the abundance of Dorea while improving the immune response of raccoon dogs by reducing the abundance of Blautia and Gemmiger.

**Discussion:**

In conclusion, MOS can improve the fur quality, serum immunity, antioxidant capacity, and gut microbiota of raccoon dogs. Therefore, MOS has the potential to replace antibiotics.

## 1 Introduction

The Chinese fur animal breeding business, particularly for raccoon dogs (*Nyctereutes procyonoides*), has grown significantly in recent years. Raccoon dogs are one of the easiest fur animals to raise, with the characteristics of short breeding cycles and strong adaptability, and their fur has extremely high economic value (Zhao et al., 2022). However, with the expansion of the breeding scale, the likelihood and extent of harmful microorganism contamination increases significantly (Liu et al., 2020). Bacterial infections have been a leading cause of mortality in the breeding of raccoon dogs. Farms often employ antibiotics to resolve this issue; however, this practice results in a high quantity of antibiotics with antibiotic residues, bacterial resistance, environmental contamination, and other undesirable consequences (Peng et al., 2019). Mannan oligosaccharides (MOS) are a critical component of the yeast cell wall. They are oligosaccharides produced by the concentration of mannose protein. Since MOS may interact with pathogens and inhibit their colonization of the gut, MOS are anticipated to become a new green feed additive in place of antibiotics (Bernal-Barragán et al., 2007). In studies related to poultry and piglets, it has been proven that dietary MOS can increase serum immunoglobulin levels, indicating that MOS can enhance animal disease resistance (Rosen, 2007). It has been reported that MOS have a beneficial effect on growth performance and nutrient digestibility in weanling pigs (Zhao et al., 2012). The apparent digestibility of nutrients determines the growth performance and the quality of the fur, with intestinal morphology playing a decisive role in the digestibility of nutrients (Cui et al., 2017). Numerous domestic and international researchers have discovered that MOS, as a prebiotic, can have beneficial biological effects on the gut by improving the composition and activity of the gut microbiota (Bindels et al., 2015). The gut microbiota is closely related to digestion rate, immunity, and antioxidant capacity. However, the composition and function of the gut microbiota are dynamic and influenced by dietary characteristics (Beam et al., 2021). It has been reported that supplementing MOS in the diet can improve the intestinal mucosa barrier and regulate the gut microbiota of weaned piglets (Zha et al., 2023). From this, we speculate that MOS may improve intestinal health, antioxidant capacity, immunity, and fur quality by regulating the gut microbiota. However, the effect of MOS on the raccoon dogs has not been studied. Therefore, we investigated the effects of MOS on the fur quality, nutrient digestibility, serum immune, serum antioxidant indices, organ indices, intestinal morphology, and gut microbiome of fur-growing raccoon dogs to provide a theoretical and practical foundation for the application of MOS in high-quality fur animal breeding.

## 2 Materials and methods

### 2.1 Raccoon dogs, dietary treatments, and experimental design

Raccoon dogs (120 ± 5 d) with similar body weights (5.01 ± 0.52 kg) were purchased from Zhang Guannian Fox and Raccoon Breeding Farm in Jilin Province, China. A total of 24 male raccoon dogs were randomly assigned to four treatment groups (*n* = 6). Each raccoon dog was kept in an individual cage (1.0 × 1.0 m). Feed was provided at 07:00 and 18:00 every day. Each cage was fitted with a feeder that provided feed at a rate of 0.3 kg/raccoon dog/d and a drinker that provided water *ad libitum*. The raccoon dogs’ diets were supplemented with MOS (Bio-Mos, Alltech, Nicholasville, KY) at 0% (C group), 0.05% (L group), 0.1% (M group), and 0.2% (H group) of basal diet. The test period consisted of a 5-day acclimation phase and a 45-day feeding trial. The ingredients and chemical composition of the basal diet are presented in Table 1.

**Table 1:** Composition and nutrient levels of basal diets (DM basis, g/kg).

Ingredients	Content	Nutrient levels	Content
Extruded corn	352.00	ME/(MJ/kg)	15.23
Soybean meal	100.00	CP	25.86
Corn gluten	43.0	*CF*	19.58
Vinasse protein	200.00	Ash	6.10
Umbilical meal	100.00	Ca	1.76
Meat meal	50.00	P	1.55
Fish meal	20.00	NaCl	2.00
Blood meal	20.00	CaHPO_4_	22.50
Feather meal	20.00	Lysine	6.00
Soybean oil	60.00		
Premix	4.50		

ME, energy; CP, crude protein; CF, crude fiber. The premix provided the following per kg of diets: VA 800000IU, VD_3_ 1 800000IU, VE 5700 IU, VB_1_ 620 mg, VB_2_ 850 mg, VK_3_ 90 mg, VC 38,000 mg, nicotinic acid 4,200 mg, pantothenic acid 75 mg, choline 35 g, Fe 8,000 mg, Mn 1,000 mg, Zn 4,800 mg, I 55 mg, Se 18 mg, and Co 53 mg. ME was a calculated value, while the others were measured values.

### 2.2 Nutrient digestibility

During the last 5 days of the experiment, we recorded daily food intake and fecal weight and then stored fecal and feed samples at −20°C. For nitrogen analysis, daily samples of 3% of total fecal production were collected and kept in wide-mouth vials containing 20 mL of 10% H_2_SO_4_. At the end of the data collecting period, the fecal and feed samples were thawed and pooled and then dried in a forced-air oven at 65°C for 72 h. In a fodder grinder, dried pooled samples of fecal and feed were crushed through a 1-mm screen. To determine the nutritional content of the fecal and feed samples, routine chemical tests were performed following the AOAC guidelines (Feldsine et al., 2002). Briefly, the dry matter (DM) content of samples was determined by drying them to a constant weight at 105°C. After 8 h of combustion at 550°C, the ash content was measured. Organic matter (OM) was determined by the amount of weight lost after ashing. The nitrogen content was measured using the Kjeldahl procedure with CuSO_4_ as a catalyst, and the crude protein (CP) concentration was estimated using the Accepted Manuscript N 6.25 formula. The ether extract (EE) in feces and feed was determined using the diethyl ether extraction–submersion method (Feldsine et al., 2002).

### 2.3 Fur quality and organ indices

On the last day of the experiment, the raccoon dogs were humanely slaughtered via electric shock according to the guidelines outlined in the Welfare of Animals Kept for Fur Production. The skin weight, skin length, underfur length (UL), and guard hair length (HL) were measured. The weights of the thymus, pancreas, liver, spleen, and kidney were recorded, and the indices of the organ were calculated. The calculation formula of the organ indices is as follows: organ indices = organ weight (kg)/body weight (kg).

### 2.4 Serum immune and antioxidant indices

At 45 d of feeding trial, after 6 h of feed withdrawal, blood samples were collected from the hind limb of three raccoon dogs per treatment. We adjusted the centrifuge (Sigma, Germany) speed to 5,000 × *g*, centrifuged the blood for 15 min, and then took the serum. Immunoglobulin A (IgA), immunoglobulin G (IgG), immunoglobulin M (IgM), catalase (CAT), and superoxide dismutase (SOD) of serum were detected by using commercial kits (Jiangsu Meimian Industrial Co., Ltd., Yancheng, China). The results were measured at 450 nm on a spectrophotometer (Shanghai Spectrophotometer Co., Ltd., Shanghai, China).

### 2.5 Intestinal morphology

On the last day of the experiment, we collected 2–3 cm of the duodenum, ileum, and jejunum and placed them in 10% formalin solution for 48 h. We adjusted the slice thickness to 5 μm and stained the sections using hematoxylin and eosin. The slices were determined using 40 × magnification (Olympus, Tokyo, Japan), and Image-Pro Plus 6.0 software (Media Cybernetics, Rockville, MD) was employed to measure the villus height (VH), crypt depth (CD), and the ratio between them at three different positions on each slice.

### 2.6 Gut microbiome

On the 45th day of the feeding trial, three raccoon dogs were randomly selected from each group to collect cecal contents after euthanasia and placed in sterile tubes containing 20% (wt/vol) maltodextrin. After flash freezing in liquid nitrogen, the tubes were lyophilized and kept at −80°C for future examination. According to the manufacturer’s instructions, we used the Qiagen magnetic bead extraction kit (Qiagen, Valencia, California, USA) to extract the total bacterial DNA from the content. Sequencing primers F (ACTCCTACGGGAGGCAGCA) and R (GGACTACHVGGGTWTCTAAT) were used to amplify the V3-V4 region of the bacterial 16S rRNA gene. Then, sequencing was performed on the Illumina NovaSeq 6,000 platform to generate paired-end reads, and the end reads were merged into the original labels. Bioinformatics analysis of the microbiome was conducted using QIME 22019 to obtain high-quality clean tags. The tags were compared to the SILVA database (version 138), and the DADA2 plugin was then used to filter the sequences for quality and to denoise them, combine them, and eliminate chimera (Callahan et al., 2016). The UPARSE algorithm (UPARSE version 7.0.1001) was used to cluster the effective tags from all samples, and the sequence was clustered into operational classification units (OTUs). OTU abundance information was normalized using a standard sequence number corresponding to the sample with the fewest sequences (McDonald et al., 2012). Subsequent analysis of alpha diversity and beta diversity was performed based on this output normalized data (Lozupone and Knight, 2005). The correlation between fur quality or serum indices and gut microbiota was analyzed by Spearman correlation analysis.

### 2.7 Statistical analysis

All graphs were generated using GraphPad Prism version 8 and Adobe Illustrator 2022. Statistical analysis was performed using the general linear model procedure of SPSS (22.0, IBM Co. Limited, Chicago, USA). The Shapiro–Wilk normality test and normal Q-Q plots were used for the normality test. Data were averaged into one value per animal and analyzed by one-way analysis of variance (ANOVA). Differences between groups were assessed using Duncan’s test. Data are presented as the mean and standard deviation (mean ± SD). A significance level of *p* < 0.05 was considered statistically significant, while *p* > 0.05 was considered not statistically significant. STAMP software (*t*-test) was used to analyze the differences in microbiota abundance between groups, and the Benjamini–Hochberg FDR multiple test correction method was used to control the false positive rate.

## 3 Results

### 3.1 Fur quality

The fur quality is shown in Table 2. It can be seen that compared to the control group, adding 0.05 and 1% MOS to the basal diet significantly increased UL and GL (*p* < 0.05). Final body weight, skin weight, and skin length were not significantly different among the groups (*p* > 0.05).

**Table 2:** Effects of MOS on fur quality of raccoon dog (%).

Parameter	C (0% MOS)	L (0.05% MOS)	M (0.1% MOS)	H (0.2% MOS)
Final body weight (kg)	6.88 ± 0.26	7.17 ± 0.56	7.33 ± 0.47	7.05 ± 0.26
Skin weight (kg)	1.98 ± 0.22	2.10 ± 0.28	2.17 ± 0.31	2.44 ± 0.54
Skin length (cm)	79.50 ± 2.88	77.83 ± 3.71	83.00 ± 4.42	79.17 ± 5.42
Underfur length (UL) (cm)	6.82 ± 0.59^b^	8.08 ± 0.86^a^	8.00 ± 0.79^a^	6.83 ± 0.93^b^
Guard hair length (GL) (cm)	8.80 ± 0.51^b^	11.00 ± 0.84^a^	10.40 ± 0.89^a^	8.95 ± 1.43^b^

UL, underfur length; GL, guard hair length. Fur-growing raccoon dogs were fed 0% (C), 0.05% (L), 0.1% (M), and 0.2% (H) MOS. The values in the table are means ± SD. ^a, b^ Means in the same row; there were significant differences in different superscripts (*p* < 0.05); no letter or with the same superscripts means no significant differences (*p* > 0.05).

### 3.2 Apparent nutrient digestibility

The apparent nutrient digestibility is shown in Table 3. The addition of 0.05 and 0.1% MOS in the diet increased apparent CP digestibility (*p* < 0.05). The dietary treatments had no significant effects on the apparent digestibility of DM, EE, and OM (*p* > 0.05).

**Table 3:** Effects of MOS on apparent nutrient digestibility of raccoon dog (%).

Parameter	C (0% MOS)	L (0.05% MOS)	M (0.1% MOS)	H (0.2% MOS)
DM	81.83 ± 3.88	84.48 ± 4.93	85.86 ± 2.27	86.33 ± 6.70
CP	73.10 ± 2.84^b^	77.76 ± 2.87^a^	75.78 ± 3.83^a^	73.78 ± 3.79^ab^
EE	89.76 ± 2.91	89.50 ± 3.48	85.48 ± 4.72	85.78 ± 7.50
OM	84.50 ± 3.43	88.63 ± 3.42	86.80 ± 4.18	86.71 ± 3.87

DM, dry matter; CP, crude protein; EE, ether extract; OM, organic matter. Fur-growing raccoon dogs were fed 0% (C), 0.05% (L), 0.1% (M), and 0.2% (H) MOS. The values in the table are means ± SD. ^a, b^ Means in the same row; there were significant differences in different superscripts (*P* < 0.05); no letter or with the same superscripts means no significant differences (*p* > 0.05).

### 3.3 Serum immune and antioxidant indices

The dietary MOS significantly affected the antioxidant and immunity capacity of the raccoon dogs (*p* < 0.05). As shown in Table 4, compared to the control group, adding 0.05% MOS significantly increased the level of CAT (*p* < 0.05). Meanwhile, adding 0.05 and 1% MOS to the basal diet significantly increased the level of IgA and IgG in the serum (*p* < 0.05). At the same time, adding different levels of MOS in the basal diet significantly increased serum IgM and SOD compared to the control group (*p* < 0.05).

**Table 4:** Effects of MOS on serum immune and antioxidant indices of raccoon dog (%).

Parameter	C (0% MOS)	L (0.05% MOS)	M (0.1% MOS)	H (0.2% MOS)
IgA/(ug/ml)	3.87 ± 0.24^b^	4.72 ± 0.43^a^	4.53 ± 0.09^a^	4.23 ± 0.16^ab^
IgG/(g/l)	3.21 ± 0.40^b^	3.77 ± 0.12^a^	3.83 ± 0.21^a^	3.50 ± 0.79^ab^
IgM/(g/l)	1.58 ± 0.15^b^	3.44 ± 0.74^a^	3.97 ± 0.26^a^	3.03 ± 0.93^a^
CAT/(U/mL)	38.76 ± 5.44^b^	56.41 ± 2.49^a^	46.46 ± 4.90^ab^	44.98 ± 2.15^ab^
SOD/(U/mL)	2.69 ± 0.41^b^	4.41 ± 0.11^a^	4.47 ± 0.15^a^	4.64 ± 0.20^a^

IgA, immunoglobulin A; IgG, immunoglobulin G; IgM, immunoglobulin M; CAT, catalase; SOD, superoxide dismutase. Fur-growing raccoon dogs were fed 0% (C), 0.05% (L), 0.1% (M), and 0.2% (H) MOS. The values in the table are means ± SD. ^a, b^ Means in the same row; there were significant differences in different superscripts (*P* < 0.05); no letter or with the same superscripts means no significant differences (*P* > 0.05).

### 3.4 Organ indices

The organ indices are shown in Table 5. It can be seen that adding 0.2% MOS in the basal diet significantly increased the indices of the thymus (*p* < 0.05), but there were no significant differences in the indices of the pancreas, liver, spleen, and kidney among the groups (*p* > 0.05).

**Table 5:** Effects of MOS on organ indices of raccoon dog.

Parameter	C (0% MOS)	L (0.05% MOS)	M (0.1% MOS)	H (0.2% MOS)
Thymus	0.04 ± 0.03^b^	0.05 ± 0.02^ab^	0.06 ± 0.02^ab^	0.09 ± 0.01^a^
Pancreas	0.14 ± 0.01	0.12 ± 0.02	0.09 ± 0.04	0.15 ± 0.01
Liver	3.60 ± 0.21	3.84 ± 0.24	4.02 ± 1.14	4.32 ± 0.44
Spleen	0.25 ± 0.11	0.31 ± 0.14	0.29 ± 0.06	0.17 ± 0.06
Kidney	0.56 ± 0.02	0.61 ± 0.01	0.75 ± 0.19	0.71 ± 0.06

Fur-growing raccoon dogs were fed 0% (C), 0.05% (L), 0.1% (M), and 0.2% (H) MOS. The values in the table are means ± SD. ^a, b^ Means in the same row; there were significant differences in different superscripts (*P* < 0.05); no letter or with the same superscripts means no significant differences (*P* > 0.05).

### 3.5 Intestinal morphology

As shown in Table 6, compared to the control group, adding 0.05 and 0.1% MOS significantly increased VH/CD of the duodenum, while 0.1% MOS increased VH of the duodenum (*p* < 0.05). Adding 0.05% MOS in the basal diet significantly increased VH/CD of the ileum and VH of the jejunum compared to the control group (*p* < 0.05). Adding 0.05% MOS significantly increased the CD of the jejunum compared to 0.2% MOS (*p* < 0.05). There were no significant differences in the CD of the duodenum, VH of the ileum, CD of the ileum, and VH/CD of the jejunum among the groups (*p* > 0.05).

**Table 6:** Effects of MOS on intestinal morphology of Ussuri raccoon dog.

Parameter	C (0% MOS)	L (0.05% MOS)	M (0.1% MOS)	H (0.2% MOS)
Duodenum				
VH (μm)	580.97 ± 79.92^b^	735.87 ± 132.19^b^	1232.79 ± 159.28^a^	749.91 ± 154.28^b^
CD (μm)	338.19 ± 95.76	235.25 ± 101.63	394.38 ± 156.80	310.61 ± 92.11
VH/CD	2.06 ± 0.60^b^	3.90 ± 0.62^a^	3.51 ± 0.44^a^	2.72 ± 0.44^b^
Ileum				
VH (μm)	1077.40 ± 373.07	1474.32 ± 656.76	853.67 ± 109.62	1013.50 ± 490.90
CD (μm)	296.83 ± 120.96	333.79 ± 131.24	249.21 ± 88.77	258.35 ± 116.93
VH/CD	3.76 ± 0.66^b^	4.32 ± 0.29^a^	3.10 ± 0.29^b^	3.66 ± 0.53^b^
Jejunum				
VH (μm)	737.80 ± 169.40^b^	1366.69 ± 305.71^a^	706.16 ± 120.09^b^	714.51 ± 37.92^b^
CD (μm)	234.53 ± 56.9^ab^	286.75 ± 109.95^a^	200.19 ± 42.45^ab^	138.84 ± 55.71^b^
VH/CD	3.54 ± 1.07	4.89 ± 0.52	3.62 ± 0.93	4.42 ± 0.60

VH, villus height; CD, crypt depth. Fur-growing raccoon dogs were fed 0% (C), 0.05% (L), 0.1% (M), and 0.2% (H) MOS. The values in the table are means ± SD. ^a, b^ Means in the same row; there were significant differences in different superscripts (*P* < 0.05); no letter or with the same superscripts means no significant differences (*P* > 0.05).

### 3.6 Gut microbiota

**Figure 1:**
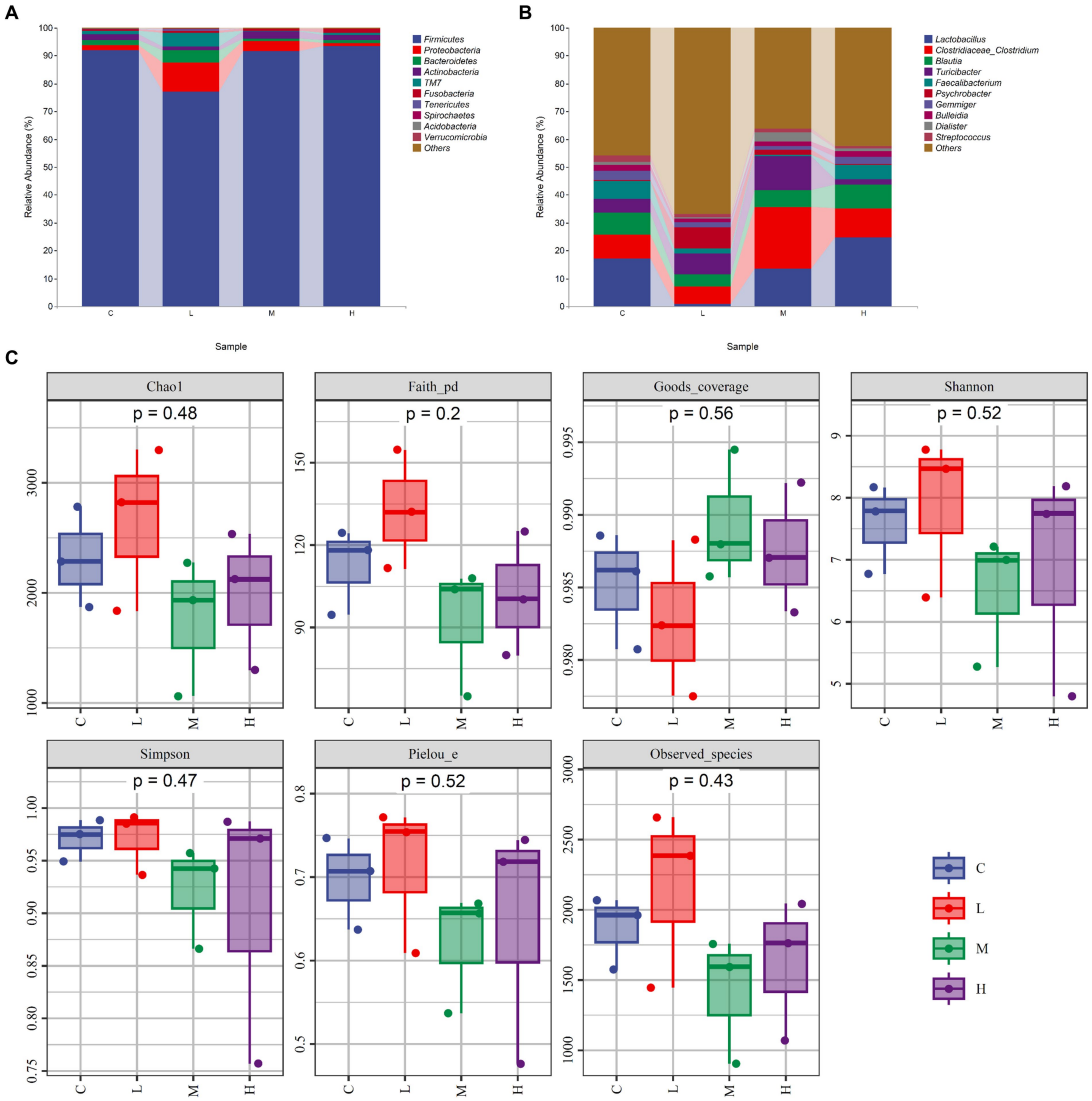
The composition and α-diversity of gut microbiota. **(A)** Relative abundance of bacteria at the phylum level. **(B)** Relative abundance of bacteria at the genus level. **(C)** α-diversity—Chao1, Faith, Goods, Shannon, Simpson, Pielou, and Observed. Fur-growing raccoon dogs were fed 0% (C), 0.05% (L), 0.1% (M), and 0.2% (H) MOS.

**Figure 2:**
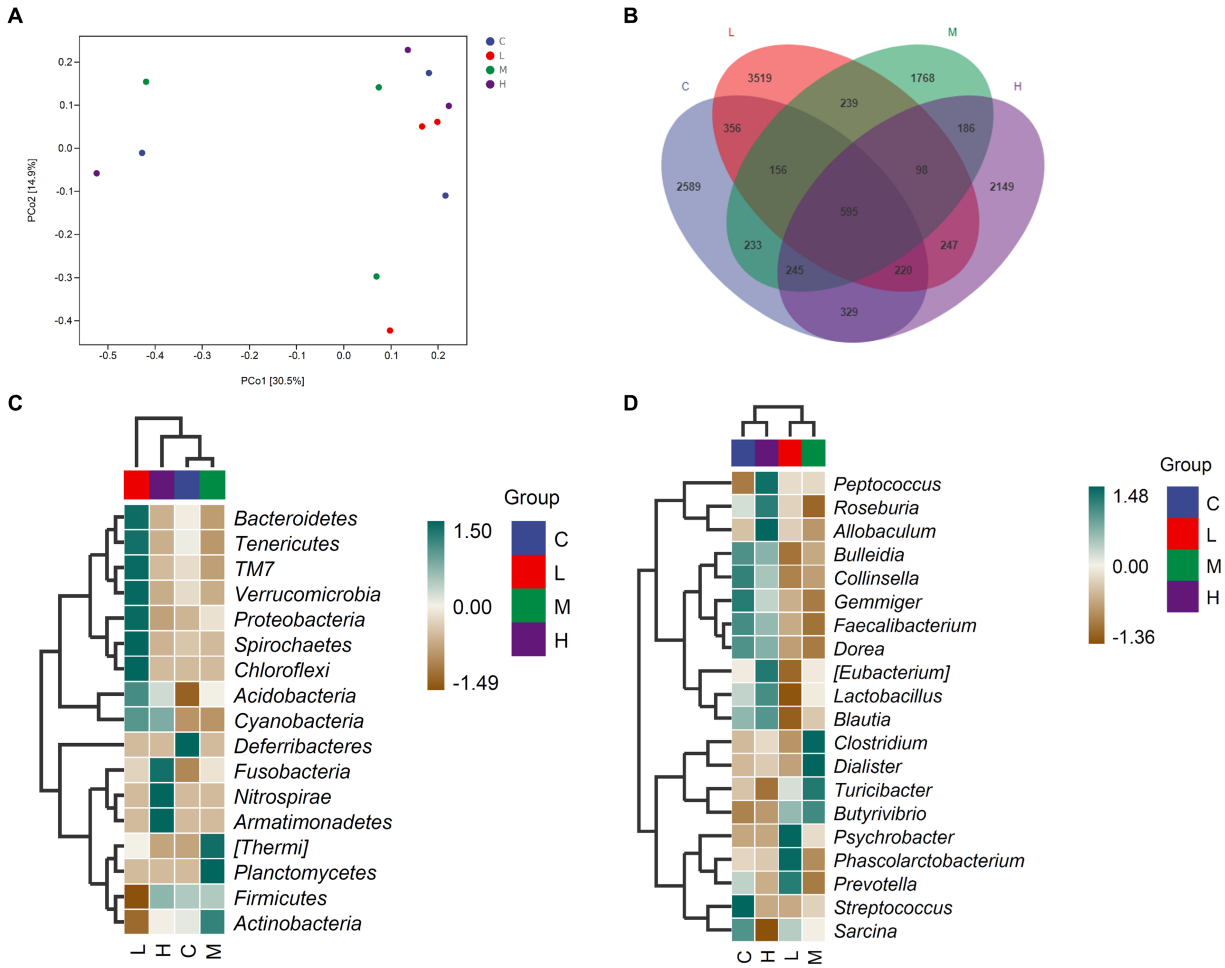
The comparison of gut microbiota among the groups. **(A)** PCoA analysis, **(B)** Venn diagram analysis, **(C)** Significant differences in bacteria at the phylum level, **(D)** Significant differences in bacteria at the genus level. Fur-growing raccoon dogs were fed 0% (C), 0.05% (L), 0.1% (M), and 0.2% (H) MOS.

The relative abundance of gut microbiota was calculated at the phylum and genus levels (Figures 1A,B). The top 10 species at the phylum level and the top 15 species at the genus level were chosen for study based on the findings of the species annotation. As shown in Figure 1A, the most dominating bacteria in the cecum of the raccoon dogs were Firmicutes, Proteobacteria, Bacteroidetes, Actinobacteria, TM7, Fusobacteria, Tenericutes, Spirochaetes, Acidobacteria, Verrucomicrobia, and others. The dominating bacteria at the genus level were *Lactobacillus*, *Clostridiaceae_Clostridium*, *Blautia*, *Turicibacter*, *Faecalibacterium*, *Psychrobacter*, *Gemmiger*, *Bulleidia*, *Dialister*, *Streptococcus*, and others. To explore α-diversity, we conducted Chao1, Faith, Goods, Shannon, Simpson, Pielou, and Observed analysis on the four groups of data (Figure 1C). The study results showed that different concentrations of MOS treatment had no significant impact on α-diversity (*p* > 0.05). To ascertain the degree of similarity across the groups, principal coordinate analysis (PCoA) plots of the Bray–Curtis distance, a representative measure of β-diversity, were created. As shown in Figure 2A, there was no significant difference in the diversity among the groups (*p* > 0.05). Venn diagram analysis was used to obtain a better understanding of the common richness across all groupings. As shown in Figure 2B, the L group included 3,519 unique OTUs. In addition, group C, M, and H included 2,589, 1768, and 2,149 unique OTUs, respectively. To further compare the differences in species composition between the samples, we plotted heat maps based on abundance at the phylum and genus levels (Figures 2C,D). The dominating bacteria at the phylum level were Bacteroidetes, Tenericutes, TM7, Verrucomicrobia, Proteobacteria, Spirochaetes, Chloroflexi, Acidobacteria, Cyanobacteria, Deferribacteres, Fusobacteria, Nitrospirae, Armatimonadetes, Thermi, Planctomycetes, Firmicutes, and Actinobacteria. At the genus level, *Peptococcus*, *Roseburia*, *Allobaculum*, *Bulleidia*, *Collinsella*, *Gemmiger*, *Faecalibacterium*, *Dorea*, *Eubacterium*, *Lactobacillus*, *Blautia*, *Clostridium*, *Dialister*, *Turicibacter*, *Butyrivibrio*, *Psychrobacter*, *Phascolarctobacterium*, *Prevotella*, *Streptococcus*, and *Sarcina* were the differential bacteria among the groups.

### 3.7 Correlation between fur quality or serum indices and gut microbiota

**Figure 3:**
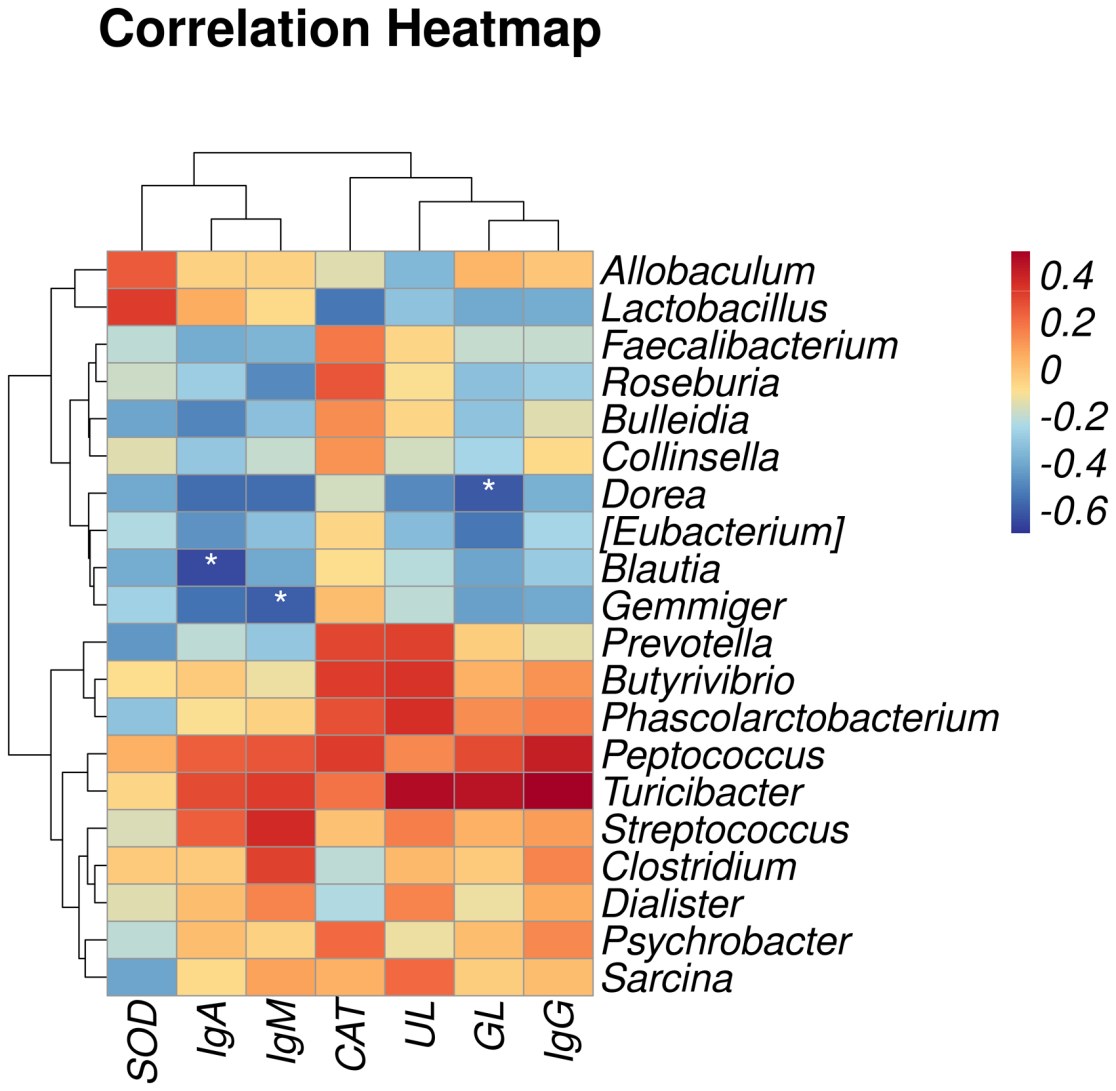
Heatmap of correlation between fur quality or serum indices and gut microbiota. SOD, superoxide dismutase; IgA, immunoglobulin A; IgM, immunoglobulin M; CAT, catalase; UL, underfur length; GL, guard hair length. IgG, immunoglobulin G. The results of the correlation analysis were analyzed by Spearman correlation analysis; *p < 0.05.

Spearman’s rank correlation analysis was performed to evaluate the correlation between fur quality or serum indices and gut microbiota. As shown in Figure 3, *Dorea* was negatively correlated with GL, *Blautia* was negatively correlated with IgA, and *Gemmiger* was negatively correlated with IgM (*p* < 0.05).

## 4 Discussion

This study aimed to evaluate the effects of MOS on the fur quality, nutritional apparent digestibility, serum parameters, intestinal morphology, and gut microbiota of raccoon dogs. As a type of fur animal, the fur quality of raccoon dogs determines economic benefits. MOS are extracted from the total amount of inactivated brewing yeast, which has potential prebiotic effects. MOS can enhance the activity of immunoglobulin, regulate intestinal inflammatory response, and promote fur growth. However, there are few reports on the effect of MOS on fur animals. The results of the present study indicated that the beneficial effects of MOS on fur quality and serum indices of raccoon dogs may be caused by regulating the gut microbiota.

The digestibility of nutrients is critical for animal development. This study found that adding 0.05 and 1% MOS to the diet could significantly increase the apparent digestibility of CP in fur-growing raccoon dogs. Additionally, when the MOS level rose, the dry matter digestibility improved. This is consistent with the findings of earlier tests. Salavati et al. (2021) showed that supplementing broiler chicken diets with 100 mg/kg MOS improved the digestibility and utilization of CP (Salavati et al., 2021). Akter et al. (2016) also discovered that adding MOS to the diet improved the feed coefficient, feeding rate, CP apparent digestibility, and total protein content of juvenile striped catfish to varying degrees, and the entire dataset demonstrated a positive correlation trend (Akter et al., 2016). MOS can improve pig feed efficiency by improving intestinal health and regulating the colonization of the gut microbiota (Wenner et al., 2013). Adding 2 or 4 g/kg MOS to the diet of piglets can improve the digestibility of CP in the ileum (Nochta et al., 2010). Similarly, MOS at 1 g/kg of diet can improve the digestion rate of rabbits by regulating the microbial population in the cecum (Bovera et al., 2012). It can be seen that supplementing MOS in the diet had a positive effect on the digestibility of CP under the conditions of this study. In addition, the intestinal morphology also determined the digestibility of CP.

Intestinal morphology is the basis for intestinal functions and health. Because the gut is an essential digestive organ of animals, and a healthy gut is required for effective food digestion, a healthy intestinal is critical for animal growth and development (Kogut and Arsenault, 2016). The structure of the small intestine is directly related to its digestive ability, and the ratio of VH to CD is a key indicator of the digestive ability of the small intestine (Wang et al., 2020). Recent studies have found a positive correlation between the VH and the expression of nutrient transport proteins. When the intestinal villi are damaged, this can damage the digestive and absorptive capacity of intestinal nutrients, and improving intestinal morphology is an important way to improve the growth performance and health of weaned piglets (Yi et al., 2021). Our study has found that MOS can improve the VH of the duodenum, VH of the jejunum, and VH/CD of the duodenum and ileum of raccoon dogs. This is similar to our results demonstrating that MOS enhanced digestibility by lengthening and expanding the surface area of villi and decreasing the CD. Adding 0.04% MOS to broiler diet can increase the VH of the jejunum on day 35, and the benefits of MOS for the intestinal morphology may be attributed to its regulatory effect as a prebiotic on the intestinal microbiota and the intestinal environment (Teng et al., 2021). The positive effect of MOS on the intestinal morphology may be an important reason for improving the apparent digestibility of CP and thereby improving fur quality.

Our study found that the inclusion of 0.05 and 0.1% MOS to the diets of fur-growing racoon dogs could enhance UL and GL, leading to an improvement in fur quality. Currently, research on oligosaccharides promoting hair growth is gradually increasing. As a type of oligosaccharide, algal oligosaccharides can restore dihydrotestosterone-induced changes in dermal papillae cell regulatory factors, thereby improving androgenetic alopecia to some extent, possibly through its antioxidant activity (Jin et al., 2020). Similarly, our study also found that MOS can enhance the antioxidant capacity of raccoon dogs. In addition, a study has found that biological oligosaccharides can stimulate *de novo* regeneration of robust hairs by regulating immune responses (Huang et al., 2022). These findings suggest that the improvement effect of MOS on the quality of raccoon dog fur may be through enhancing antioxidant capacity and immune response pathways.

The adaptive immune system is composed of T and B lymphocytes, with the thymus being the main lymphatic organ necessary for the development of T lymphocytes, providing monitoring and protection for the body’s defense mechanisms (Thapa and Farber, 2019). Our study found that supplementing 0.2% MOS to the diet of raccoon dogs could increase the thymus indices. Similarly, adding 0.5 g/kg MOS to the diet could increase the weight of the thymus and promote the health and immune response of broiler chicks (Attia et al., 2017). Taken together, it can be seen that MOS has a positive effect on promoting the growth of the thymus gland and maintaining the immune system.

According to earlier research, MOS may connect to immune cell-related receptors. This process activates the immune defense system, increases the generation of cytokines, enhances the capacity of antigen-presenting cells to deliver antigens, and strengthens the body’s non-specific immunity. Among these, the MOS binding protein plays a critical role in non-specific immunity. After MOS stimulate the liver effectively, the MOS binding protein may be generated. The MOS binding protein, as a calcium-dependent coagulant, may initiate the complement cascade enzyme process, causing the body to generate an immunological response (Patel et al., 2015). Additionally, toll-like receptors are capable of recognizing and binding MOS. After this process is completed successfully, ligand-receptor complexes may be created to control the synthesis of receptor-related signal protein molecules and the activation of signal pathways in immune cells, inducing the release of cytokines and initiating the immunological response (Nikolakopoulou and Zarkadis, 2006). Immunoglobulin is an important component of humoral immunity, and the content of immunoglobulin in the blood may be a clear indicator of the strength of the body’s immune system (Han et al., 2018). IgG is the most abundant antibody in animals, constituting 75% of total immunoglobulin in serum and performing antiviral, antibacterial, and immunological regulatory activities (Navolotskaya, 2014). IgM is not only a critical component of the basal immune response, but it is also the first antibody subtype seen throughout the infectious immune response process. IgA is a non-inflammatory antibody that exists naturally in the body and is the primary component of the mucosal defense system. Our results indicated that adding different levels of MOS to the diet can improve the immune function of fur-growing raccoon dogs. It has also been found that adding MOS to goat milk can regulate the serum IgG content of newborn goats (Yang et al., 2022). Meanwhile, Zhou et al. (2019) discovered that supplementing broiler feed with MOS substantially increases IgG and IgM levels (Zhou et al., 2019). The increase in IgA, IgM, and IgG in serum indicates that MOS have a positive effect on promoting immune response. They are capable of resisting harmful germs’ invasion, inhibiting viral reproduction, and acting as an immunological barrier. Recent studies have increasingly revealed the vital impact of the local immune microenvironment on hair growth, and oligosaccharide biomaterials can promote hair growth by regulating immune responses (Huang et al., 2022). The findings indicated that supplementing the feed with MOS may significantly increase the immunoglobulin activity in the serum of fur-growing raccoon dogs to improve its immunity, which may be an important reason for promoting the quality of raccoon dog fur.

Research has found that fur quality is related to antioxidant capacity. The use of functional antioxidants and barrier enhancers to further improve scalp condition can enable a reduction in hair shedding and thus an increase in perceived hair fullness (Davis et al., 2021). Animals generate reactive oxygen species (ROS) and free radicals during metabolism and in stressful environments, and their buildup results in the breakdown of organisms’ cellular structure and function. SOD and CAT are found in all species and serve a critical role in preventing oxidative damage to cells. Their activities may be used to assess an animal’s antioxidant capability to a certain degree (Yousef et al., 2009). MOS have a natural antioxidant action, activating the antioxidant enzyme system, increasing the activity of SOD and CAT, removing excess free radicals and ROS from animals, and protecting them from oxidative damage (Zhang et al., 2015). Our study results indicated that adding 0.05% MOS to the diets can increase CAT activity while adding 0.05, 0.1, and 0.2% MOS can increase SOD activity. Zheng et al. (2018) discovered that MOS may substantially increase the antioxidant capacity of sheep, particularly at a dosage of 1.6% (Zheng et al., 2018). Attia et al. (2017) discovered that intermittent addition of MOS in broiler diets may substantially increase SOD activity (Attia et al., 2017). The findings of this experiment were mostly similar to those of the previous experiment, indicating that supplementing MOS in the diet may improve the fur quality of raccoon dogs by enhancing their antioxidant capacity.

To further investigate the impact of MOS on the fur quality and serum indices of raccoon dogs, we recreated their natural breeding habitat and examined their gut microbiota. It has been reported that the gut microbiota is closely related to the growth, development, and production benefits of animals (Zhao et al., 2022). The gut microbiota of carnivore animals such as raccoon dogs has been reported to mainly be composed of Firmicutes, Bacteroidetes, Actinobacteria, and Proteobacteria (Liu et al., 2021; Nan et al., 2021), similarly to our results. In the present study, MOS supplementation did not affect the gut microbiota richness and diversity. A recent study also reported that MOS failed to modify the diversity and richness of gut microbiota (Kazlauskaite et al., 2022). It can be speculated that MOS will not have a significant impact on the stability of the gut microbiota. *Peptococcus* plays a key role in promoting immunity while also promoting digestion and nutrient absorption (Yang et al., 2021). Another study found that *Peptococcus* has a positive effect on improving the overall health of chickens (Zhu et al., 2016). Bacteroidetes are the main microorganisms in the intestinal tract of animals, but they may induce inflammation and promote the occurrence of host diseases (Stojanov et al., 2020). Our study results indicated that different levels of MOS can reduce the relative abundance of Bacteroidetes while increasing *Peptococcus*. Similarly, dietary supplementation with *Cyberlindnera jadinii* can improve the growth performance, serum immunity, and antioxidant capacity of growing raccoon dogs while reducing the Bacteroidetes (Zhao et al., 2022). Probiotics not only promote the growth performance and intestinal immunity of piglets but also increase the abundance of *Peptococcus* (Xin et al., 2020). The increase in *Peptococcus* and the decrease in Bacteroidetes may indicate an improvement in the gut microbiota, which has a positive effect on promoting intestinal health, antioxidant capacity, and immunity.

We investigated the effects of gut microbiota on the immunoglobulin content, antioxidant indices, and fur quality of raccoon dogs through Spearman correlation analysis. The study results showed that the relative abundance of *Dorea* was negatively correlated with GL, the abundance of *Blautia* was negatively correlated with IgA, and the relative abundance of *Gemmiger* was negatively correlated with IgM. This is similar to previous reports, where IgA levels in the serum and urine of patients with IgA nephropathy increased, while 16 s rDNA analysis showed a decrease in *Blautia* (Tang et al., 2022). Similarly, it has been reported that as a natural antioxidant polyphenol, taxifolin can increase the secretion of IgA, IgG, and IgM in dextran sulfate sodium and reduce the abundance of *Gemmiger* (Hou et al., 2021). In addition, a study found an increase in IgM content in the serum of major depressive disorder (Kling et al., 2006). Further analysis of its fecal microbiota revealed a decrease in *Gemmiger* abundance, indicating a negative correlation between IgM and *Gemmiger* (Maes et al., 2023). We speculate that MOS may enhance the immune response of raccoon dogs by reducing the relative abundance of *Blautia* and *Gemmiger*. Previous studies have found that hair follicle immune imbalance is the main pathogenic event of alopecia areata. Patients with alopecia areata can cause an increase in the abundance of *Dorea* in fecal samples (Sánchez-Pellicer et al., 2022). It can be seen that adding MOS to diets could promote the fur quality of raccoon dogs by decreasing the abundance of *Dorea*.

The results of the current study suggest that supplementing 0.05 and 1% MOS in the diets of racoon dogs can improve their gut microbiota. Therefore, MOS may play an active role in fur quality and serum indices by regulating gut microbiota, and this effect was greater when the addition level was 0.05 or 0.1%. However, due to the small number of raccoon dogs available for the study under the actual conditions of the experiment, further validation of the results is required.

## 5 Conclusion

In conclusion, adding 0.05 and 1% MOS to the diets of racoon dogs can improve the apparent digestibility of CP and intestinal morphology. In addition, MOS can also be used to optimize their gut microbiota structure. MOS can improve the fur quality of racoon dogs by reducing the abundance of *Dorea* while improving their immune response by reducing the abundance of *Blautia* and *Gemmiger*. Therefore, MOS have potential as an efficient antibiotic alternative in raccoon dog feed.

## Data availability statement

The datasets presented in this study can be found in online repositories. The names of the repository/repositories and accession number(s) can be found in the article/Supplementary Material.

## Ethics statement

The animal studies were approved by Jilin Agricultural University in P.R. China and approved by the Experimental Animal Welfare and Ethics Committee of Jilin Agricultural University. The studies were conducted in accordance with the local legislation and institutional requirements. Written informed consent was obtained from the owners for the participation of their animals in this study.

## Author contributions

CY: Writing – original draft. LR: Software, Writing – original draft. RS: Methodology, Writing – original draft. XY: Methodology, Writing – original draft. XZ: Methodology, Writing – original draft. AZ: Supervision, Writing – review & editing. MW: Project administration, Writing – review & editing.
